# Fabrication of Chitosan/Silk Fibroin Composite Nanofibers for Wound-dressing Applications

**DOI:** 10.3390/ijms11093529

**Published:** 2010-09-21

**Authors:** Zeng-xiao Cai, Xiu-mei Mo, Kui-hua Zhang, Lin-peng Fan, An-lin Yin, Chuang-long He, Hong-sheng Wang

**Affiliations:** 1 Key Laboratory of Textile Science & Technology, Ministry of Education, Donghua University, Shanghai, 201620, China; E-Mail: caizengxiao@sina.com; 2 Biomaterials and Tissue Engineering Lab, College of Chemistry and Chemical Engineering and Biological Engineering, Donghua University, Shanghai, 201620, China; 3 College of Biological Engineering and Chemical Engineering, Jiaxing College, Zhejiang, 314001, China

**Keywords:** electrospinning, chitosan, composite nanofibers, antibacterial activity, wound dressing

## Abstract

Chitosan, a naturally occurring polysaccharide with abundant resources, has been extensively exploited for various biomedical applications, typically as wound dressings owing to its unique biocompatibility, good biodegradability and excellent antibacterial properties. In this work, composite nanofibrous membranes of chitosan (CS) and silk fibroin (SF) were successfully fabricated by electrospinning. The morphology of electrospun blend nanofibers was observed by scanning electron microscopy (SEM) and the fiber diameters decreased with the increasing percentage of chitosan. Further, the mechanical test illustrated that the addition of silk fibroin enhanced the mechanical properties of CS/SF nanofibers. The antibacterial activities against *Escherichia coli* (Gram negative) and *Staphylococcus aureus* (Gram positive) were evaluated by the turbidity measurement method; and results suggest that the antibacterial effect of composite nanofibers varied on the type of bacteria. Furthermore, the biocompatibility of murine fibroblast on as-prepared nanofibrous membranes was investigated by hematoxylin and eosin (H&E) staining and MTT assays *in vitro*, and the membranes were found to promote the cell attachment and proliferation. These results suggest that as-prepared chitosan/silk fibroin (CS/SF) composite nanofibrous membranes could be a promising candidate for wound healing applications.

## 1. Introduction

As a naturally occurring polysaccharide polymer, chitin is one of the most abundant natural biomaterials which can be obtained from shells of crustaceans. Chitosan is a partially *N*-deacetylated derivative of chitin, consisting of polymeric (1→4)-linked 2-amino-2-deoxy-β-d-glucopyranose units [1]. Due to the haemostatic activity, non-toxicity, biodegradability, and intrinsic antibacterial properties, chitosan and its derivatives have been used in various fields including food, agricultural, biotechnological and pharmaceutical products [2,3].

A range of different processing techniques have been developed to fabricate micro- or nano-scale fibrous matrices, including self-assembly [4], template-directed synthesis [5], phase separation [6], and electrospinning [7–10]. Among these techniques, electrospinning has been regarded as a facile and effective way to fabricate ultrafine fibers from a variety of synthetic or natural polymers [11]. Due to their high surface to volume ratio, high porosity and good inter-pore connectivity, electrospun nanofibers can be used as filter materials, sensors, wound dressings, controlled release carriers, and tissue engineering scaffolds [12].

Direct electrospinning of chitosan is absolutely difficult [13]. In order to expand the application of chitosan, electrospinning of chitosan has usually been conducted from its blend solutions with another electro-spinnable polymer, such as poly(ethylene oxide) (PEO) [14], poly(vinyl alcohol) (PVA) [13], poly(lactic acid) (PLA) [15], and silk fibroin [16]. Natural polymers are generally preceded over synthetic polymers for tissue engineering applications in the case of their proven biocompatibility and absorbable biodegradation products. Silk fibroin is extracted from Cocoons of *Bombyx mori* silkworm, which is abundant and easily gathered. Additionally, as a promising biomaterial, Silk fibroin has diverse excellent properties including good biocompatibility, remarkable air permeability and biodegradation, low inflammatory reaction and so on [17,18]. Chitosan blending with silk fibroin could facilitate the electrospinning process, and provided favorable biocompatibility and biological interactions.

Ideal wound dressings should make the wound free from infection, and have excellent biocompatibility [19]. Polymers with intrinsic bacteriostatic and/or bactericidal activity have more advantages as wound dressing materials. Therefore, the chitosan/silk fibroin membrane could be a good candidate for wound dressing applications.

The objective of this paper is to find the properties of electrospun CS/SF nanofibers, including the morphologies of CS/SF composite nanofibers, the mechanical properties, antibacterial activities and biocompatibility *in vitro*, which could be beneficial to the composite nanofibrous membranes serving as varied wound dressings.

## 2. Results and Discussion

### 2.1. Morphology of CS/SF Blend Nanofibers

**Figure 1:**
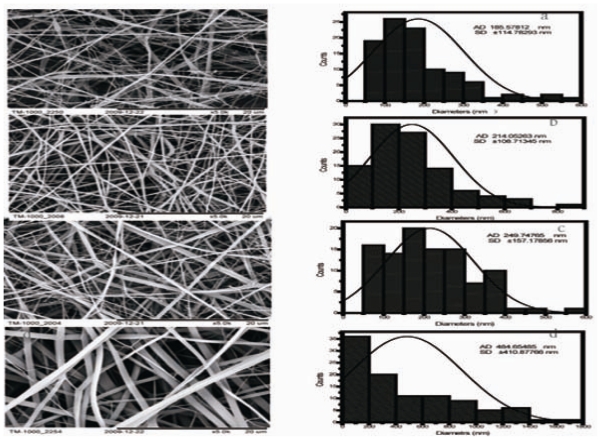
SEM micrographs of electrospun CS/SF blend fibers: (**a**) CS/SF (80/20); (**b**) CS/SF (50/50); (**c**) CS/SF (20/80); (**d**) pure SF.

The silk fibroin–chitosan composite fibers with a diameter ranging from 185.5 ± 114.7 nm to 484.6 ± 410.8 nm were fabricated using electrospinning. Figure 1 shows scanning electron microscopy (SEM) micrographs and diameter distribution histograms of CS/SF hybrid nanofibers. Pure silk fibroin nanofibers had a larger average diameter (484.6 ± 410.8 nm) than CS/SF nanofibers. The average diameters of electrospun CS/SF hybrid nanofibers were 249.7 ± 157.1 nm, 214.0 ± 108.7 nm and 185.5 ± 114.7 nm, respectively, with chitosan weight ratios of 20%, 50% and 80%. Fiber diameters decreased with the increasing of chitosan content. The other parameters involving voltage, collecting distance, feed rate and solution concentration were fixed during electrospinning, therefore, fiber diameters are mainly dependent on the ratio of chitosan to silk fibroin.

### 2.2. Crosslinking of Fibers

The nanofibers containing silk fibroin or chitosan are water soluble. Even a drop of water on the membranes can destroy the nanofibrous structure. By placing the nanofibers into a desiccator filled with GTA vapor, the CS/SF nanofibers can be crosslinked appropriately. Crosslinking of silk fibroin and chitosan with GTA involves the reaction of free amino groups of chitosan and amino acid of the silk fibroin with the aldehyde groups of GTA [23]. After crosslinking, the membranes were water insoluble.

### 2.3. Mechanical Properties

**Figure 2:**
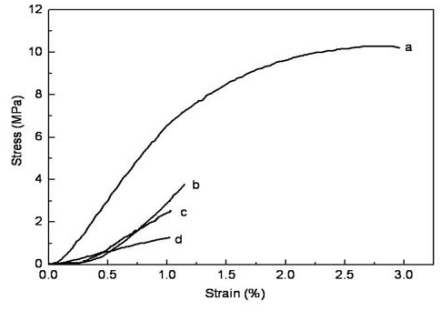
Tensile stress-strain curves of cross-linked CS/SF composite membranes with various chitosan contents. (**a**) 0%; (**b**) 20%; (**c**) 50%; (**d**) 80%.

The tensile stress-strain curves of as-prepared composite nanofibrous membranes are presented in Figure 2. The stress and strain of the composite nanofibrous membranes at break are summarized in Table 1. The tensile strength of the cross-linked nanofibrous membranes increased from 1.3 MPa to 10.3 MPa with the increased content of silk fibroin. The elongation at break of the cross-linked nanofibrous membranes showed an increased trend with the increasing proportion of silk fibroin. These results suggested that the addition of silk fibroin was beneficial to enhancing the mechanical properties of CS/SF nanofibers.

### 2.4. Evaluation of Antibacterial Activity in Vitro

**Figure 3:**
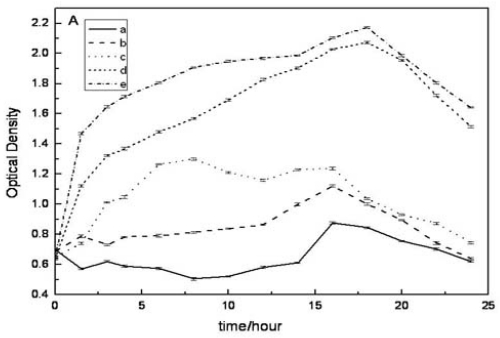
Growth curves of *E. coli* and *S. aureus* in the presence of CS/SF as measured at 570 nm. (**A**) *E. coli*; (**B**) *S. aureus* (a) CS/SF (80/20); (b) CS/SF (50/50); (c) CS/SF(20/80); (d) pure SF; (e) control growth.

In order to investigate the antibacterial activity of composite nanofibrous membranes with same weight, the optical density method was used to measure the inhibition effects of composite nanofiberous membranes with different ratios on the growth of *E. coli* and *S. aureus*. Figure 3 shows *E. coli* and *S. aureus* growth curves of the control and tested groups, respectively. The optical density (absorbance at 570 nm, A570) reflected the number of microorganisms in the cultured medium. Lower absorbance meant higher inhibition effects of composite nanofibrous membranes on the bacteria. Although the antibacterial effect on *S. aureus* had no obvious difference among those groups (Figure 3B), in Figure 3A all tested groups had less optical density compared with the control group with no nanofibrous matrices at any time point. Therefore, the composite nanofibrous membranes had obvious antibacterial effect on the *E. coli*, and the antibacterial effect increased with the increase of chitosan content. These results suggested that the content of chitosan was a dominant element in the antibacterial effect, and the antibacterial effect of composite nanofibers varied on types of bacteria.

### 2.5. Cell Morphology and Proliferation

**Figure 4:**
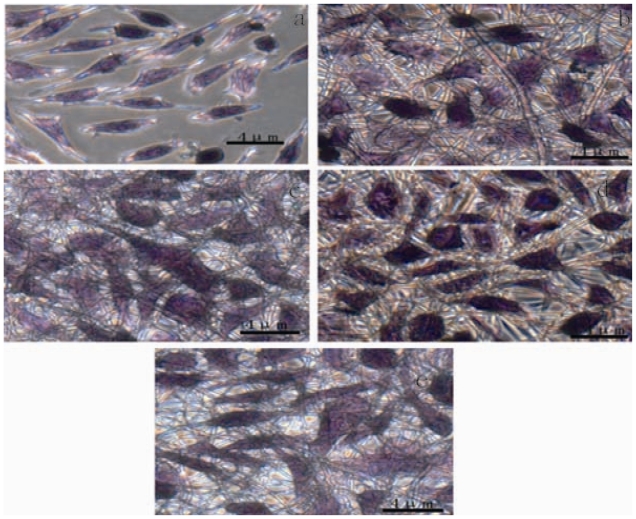
Comparison of fibroblasts proliferation on electrospun silk fibroin–chitosan nanofibers and controls: (**a**) glass cover slips; (**b**) CS/SF (80/20); (**c**) CS/SF (50/50); (**d**) CS/SF(20/80); (**e**) pure SF. (H&E Staining, ×400 light microscope).

Hematoxylin and eosin (H&E) staining was reconstructed to observing cell morphologies on the CS/SF membranes and glass cover slips, as shown in Figure 4. It can be seen that fibroblasts exhibited a spreading shape-polygonal and flatten morphology on composite nanofibrous membranes, which suggested that the cells could function biologically on the nanofibrous membranes and nanofibrous structure is available for cell attachment [24,25]. Figure 4a showed that most of the free fibroblasts on cover slips had a spread out morphology, while some fibroblasts still remained spherical in shape. The results suggested that the CS/SF composite nanofibrous membranes were beneficial to the fibroblast development.

**Figure 5:**
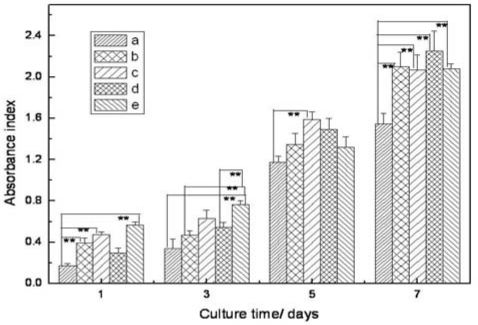
Comparison of fibroblasts proliferation on electrospun silk fibroin–chitosan nanofibers and controls: (**a**) glass cover slips; (**b**) CS/SF (80/20); (**c**) CS/SF (50/50); (**d**) CS/SF (20/80); (**e**) pure SF. Error bars represent mean ± SD for n = 3. **p < 0.01.

The level of cell growth and proliferation on these membranes and glass cover slips were assessed using MTT assay *in vitro* (Figure 5). It can be seen that the absorbance index of the tested groups increased with the increase of culture time. In the first three days, statistically significant differences (p < 0.01) were observed in the cell activity between CS/SF fibrous membranes and glass cover slips, which implied that the nanofibrous membranes were beneficial to cell development. It was found that there was no big difference in the cell activity of all the test groups except CS/SF (50/50) group after 5-day culture. While the number of fibroblasts on CS/SF fibrous membranes exhibited remarkably higher than those on glass cover slips after 7-day culture. These results indicated that different ratios of electrospun CS/SF nanofibers promoted the attachment and proliferation of fibroblasts significantly under our conditions compared to the cover slip control.

## 3. Experimental Section

### 3.1. Materials

Main CS (My Medium molecular weight; 75–85% deacetylated) was purchased from Sigma-Aldrich Chemical Company (St. Louis, Missouri, U.S.). Cocoons of Bombyx mori silkworm were kindly supplied by Jiaxing Silk Co. Ltd. (China).

The solvents used in this work include 1,1,1,3,3,3,-hexafluoro-2-propanol (HFIP, Shanghai Darui Finechemical Co., Ltd.) and 2,2,2-trifluoroethanol (TFE, Shanghai Darui Finechemical Co., Ltd.). All products were used without further purification.

### 3.2. Preparation of Regenerated SF

Raw silk fibers were boiled three times with 0.5% (w/w) NaCO3 solution for 30 min and then rinsed with distilled water. Degummed silk (silk fibroin, SF) was dissolved in a ternary solvent system of CaCl2/CH_3_CH_2_OH/H_2_O (1/2/8 in mole ratio) at 65 °C for 1 h. Then the solution was dialysed with cellulose tubular membrane (Sigma Co., 250–257 μm) in distilled water for 3 days, after filtering the regenerated SF sponges was obtained by freeze drying.

### 3.3. Electrospinning

Silk fibroin was dissolved in HFIP and Chitosan was dissolved in the mix-solvent HFIP/TFA with the volume ratio of 9/1. The two solutions were mixed together at different ratios (w/w). Electrospinning was performed as follow under room temperature. The solutions were filled into a 2.5 mL plastic syringe with a blunt-ended needle (ID = 0.21 mm). The syringe was located in a syringe pump (789100C, Cole-Parmer, U.S.) and dispensed at a rate of 0.8 mL/h. A voltage of 20 kV using a high voltage power supply (BGG6-358, BMEICO. LTD. China) was applied across the needle and ground collector, which was placed at a distance of 12–15cm.

### 3.4. Glutaraldehyde Vapor Crosslinking of Nanofibers

The CS/SF nanofibrous membrane was crosslinked in a sealed desiccator containing 10 mL of 25% glutaraldehyde (GTA) aqueous solution. The membranes were placed on a holed ceramic shelf in the desiccator and were crosslinked at room temperature for 24 hours. After crosslinking, the samples were dried by vacuum at ambient temperature.

### 3.5. Scanning Electron Microscopy Analysis

The morphologies were observed with a scanning electronic microscopy (SEM; TM-1000) at an accelerated voltage of 15 kV. The mean fiber diameters were estimated using image analysis software (Image-J, National institutes of Health, U.S.) and calculated by selecting 100 fibers randomly observed on the SEM image.

### 3.6. Mechanical Properties

Rectangular membrane specimens with a dimension of 10 mm × 50 mm were prepared according to the method reported in the literature [20,21]. The specimen thickness was exactly quantified using a micrometer with a precision of 0.01 mm. The tensile testing of membrane was performed using a universal materials tester (H5K-S, Hounsfield, U.K.) with a load cell of 50 N at ambient temperature of 20 °C and humidity of 65%. A cross-head speed of 10 mm/min was used for all of the specimens tested. Each example was measured three times. The machine-recorded data were used to plot the tensile stress–strain curves of the specimens.

### 3.7. Antibacterial Assessment

Antimicrobial activity test on membranes was carried out using turbidity measurement method. *E. coli* and *S. aureus* were taken as model gram-negative and gram-positive bacteria. The tested specimens were CS/SF (80/20), CS/SF (50/50), CS/SF (20/80), pure SF, and SF membranes with the same weight, which were sterilized for 2 h using UV.

Single colony of *E. coli* and *S. aureus* grown on agar culture medium were transferred into 100 mL of liquid seed medium separately [22]. After 12 h of cultivation at 37 °C with shaking at 160 rpm, 5 mL of cell suspension was introduced into a 250-mL Erlenmeyer flask containing 100 mL of fermentation medium, and then the membranes were put in the Erlenmeyer flask. The culture was kept at 37 °C for 24 h under agitation. Samples drawn from the systems every two hours were analyzed spectrophotometrically by measuring the absorbance at 570 nm (Spectrophotometers (UV-2102pcs, UNICO(SHANGHAI) Instruments Co., Ltd.). Studies were performed in triplicate and average values with standard deviation errors were reported.

### 3.8. Hematoxylin and Eosin (H&E) Staining and MTT Analysis

Fibroblasts (L929) were cultured in cell culture flasks with culture medium containing 10% fetal bovine serum (FBS) in RPMI 1640. The media were replaced every two days, and the culture were maintained in a humidified incubator at 37 °C with 5% CO_2_. Electrospun nanofibrous membranes were prepared on circular glass cover slips (14 mm in diameter) and fixed with steel rings in the 24-well cell culture plates. Nanofibrous membranes were sterilized by 75% ethanol for 2 h and then washed with phosphate-buffered saline solution (PBS) three times, and lastly washed with the culture media. Cells were trypsinized when their density reached 80–90% of the cell culture flask and counted with a hemocytometer.

The hematoxylin and eosin (H&E) staining was used to investigate cell attachment and proliferation. After three days incubation, the fibroblasts were fixed in 10% paraformaldehyde for 20 min, and then washed with distilled water for four minutes. After that the fibroblasts were stained in hematoxylin for 10 min, followed by washing with running tap water. The fibroblasts were then dehydrated in 95% alcohol, and the cytoplasm were stained by eosin for 2 min, then lastly dehydrated in 70% alcohol and allowed to dry. The stained fibroblasts were viewed and photographed at magnification (×400) by using Olympus phase contrast microscope (Model 1 × 70, Japan).

To evaluate cell proliferation, fibroblasts were seeded onto cover slips and nanofibrous membranes (n = 4 ) at a density of 1.1 × 10^4^ cells/well for 1, 3, 5, and 7 days, using methylthiazol tetrazolium (MTT) assay. At the appointed time the culture media were removed, and then washed three times with PBS to remove the residual culture media. Each sample was added with 360 μL serum-free DMEM medium and 40 μL MTT solution (5mg/mL MTT stock solution in PBS), and incubated at 37 °C for 4 h to allow the formation of MTT formazan. Thereafter, the culture media were extracted and 400 μL dimethylsulfoxide (DMSO) was added. When the formazan crystals were sufficiently resolved, 100 μL of each sample was poured into a 96-well plate and tested by an Enzyme-labeled Instrument (MK3, Thermo, U.S.), at 570 nm.

### 3.9. Statistical Analysis

All experiments were conducted at least three times and all values were reported as the mean and standard deviation. Statistical analysis was carried out by the one-way analysis of variance (ANOVA) performed in SPSS software. The statistical difference between two sets of data was considered when p < 0.01.

## 4. Conclusions

The best wound dressing should be biocompatible and antibacterial. In this study, blended chitosan and silk fibroin nanofibrous membranes have been successfully prepared by electrospinning in a HFIP/TFA spinning solvent. The average diameter of as-prepared nanofibers increased with the increase of silk fibroin content. Furthermore, the addition of silk fibroin enhanced the mechanical properties of composite nanofibrous membranes. From the MTT assay, it was found that CS/SF composite nanofibrous membranes promoted cell attachment and proliferation. Turbidity measurement showed the inhibition of composite nanofibrous membranes on the growth of the Gram-negative bacteria *E. coli.* The antibacterial activity increased greatly with an increasing proportion of chitosan, which was significantly beneficial to the CS/SF nanofibrous membranes serving as wound dressings. The CS/SF nanofibrous membranes could prospectively be developed as a commercial product in wound healing treatment in the future.

## Figures and Tables

**Table 1:** Mechanical Properties of Cross-Linked CS/SF composite nanofibrous membranes.

Crosslinked CS/SF (wt/wt)	0:10	2:8	5:5	8:2
Tensile stress (MPa)	10.3 ± 0.24	1.2 ± 0.13	1.1 ± 0.22	1.0 ± 0.21
Ultimate strain (%)	2.8 ± 0.22	3.8 ± 0.21	2.5 ± 0.25	1.3 ± 0.20

## References

[1] Li Y Chen XG Liu N Physicochemical characterization and antibacterial property of chitosan acetates Carbohydr. Polymer 2007 67 227 232

[2] Ignatova M Starbova K Markova N Manolova N Rashkova I Electrospun nano-fibre membranes with antibacterial properties from quaternised chitosan and poly(vinyl alcohol) Carbohydr. Res 2006 341 2098 2107 16750180 10.1016/j.carres.2006.05.006

[3] Devlieghere F Vermeulen A Debevere J Chitosan: Antimicrobial activity, interactions with food components and applicability as a coating on fruit and vegetables Food Microbiol 2004 21 703 714

[4] Hartgerink JD Beniash E Stupp SI Peptide-amphiphile nanofibers: A versatile scaffold for the preparation of self-assembling materials Proc. Natl. Acad. Sci. USA 2002 99 5133 5138 11929981 10.1073/pnas.072699999 PMC122734

[5] Hulteen JC Chen HX Chambliss CK Martin CR Template synthesis of carbon nanotubule and nanofiber arrays Nanostruct. Mater 1997 9 133 136

[6] Ma PX Zhang RY Synthetic nano-scale fibrous extracellular matrix J. Biomed. Mater. Res 1999 46 60 72 10357136 10.1002/(sici)1097-4636(199907)46:1<60::aid-jbm7>3.0.co;2-h

[7] Reneker DH Yarin AL Fong H Koombhongse S Bending instabilityof electrically charged liquid jets of polymer solutions in electrospinning J. Appl. Phys 2000 87 4531 4547

[8] Srinivasan G Reneker DH Structure and morphology of small-diameter electrospun aramid fibers Polymer Int 1995 3 195 201

[9] Reneker DH Chun I Nanometre diameter fibres of polymer, produced byelectrospinning Nanotechnology 1996 7 216 223

[10] Yarin AL Koombhongse S Reneker DH Bending instability in electrospinning of nanofibers J. Appl. Phys 2001 89 3018 3026

[11] Jayaraman K Kotaki M Zhang YZ Mo XM Ramakrishna S Recent advances in polymer nanofibers J. Nanosci. Nanotechnol 2004 4 52 65 15112541

[12] Li D Xia Y Electrospinning of nanofibers reinventing the wheel Adv. Mater 2004 16 1151

[13] Ohkawa K Cha D Kim H Nishida A Yamamoto H Electrospinning of chitosan Macromol. Rapid Commun 2004 25 1600 1605

[14] Desai K Kit K Li J Zivanovic S Morphological and surface properties of electrospun chitosan nanofibers Biomacromolecules 2008 9 1000 1006 18198844 10.1021/bm701017z

[15] Xu J Zhang J Gao W Liang H Wang H Li J Preparation of chitosan/PLA blend micro/nanofibers by electrospinning Mater. Lett 2009 63 658 660

[16] Park WH Jeong L Yoo DI Hudson S Effect of chitosan on morphology and conformation of silk fibroin nanofibers Polymer 2004 45 7151 7157

[17] Sakabe H Ito H Miyamoto T Noishiki Y Ha WS *In vivo* blood compatibility of regenerated silk fibroin Sen-i Gakkaishi 1989 45 487 490

[18] Santin M Motta A Freddi G Cannas M *In vitro* evaluation of the inflammatory potential of the silk fibroin J. Biomed. Mater. Res 1999 46 382 389 10397996 10.1002/(sici)1097-4636(19990905)46:3<382::aid-jbm11>3.0.co;2-r

[19] Purna SK Babu M Collagen based dressings–a review Burns 2000 26 54 62 10630321 10.1016/s0305-4179(99)00103-5

[20] Huang ZM Zhang YZ Ramakrishna S Lim CT Electrospinning and mechanical characterization of gelatin nanofibers Polymer 2004 45 5361

[21] Chen ZG Wei B Mo XM Mechanical properties of electrospun collagen–chitosan complex single fibers and membrane Mater. Sci. Eng. C 2009 29 2428 2435

[22] Wang X Du Y Liu H Preparation, characterization and antibacterial activity of chitosan–Zn complex Carbohydr. Polymers 2004 56 21 26

[23] Chen ZG Wang PW Wei B Mo XM Cui FZ Electrospun collagen–chitosan nanofiber: A biomimetic extracellular matrix for endothelial cell and smooth muscle cell Acta Biomater 2010 6 372 382 19632361 10.1016/j.actbio.2009.07.024

[24] Zhou Y Yang D Chen X Xu Q Lu F Nie J Electrospun water-soluble carboxyethyl chitosan/poly(vinyl alcohol) nanofibrous membrane as potential wound dressing for skin regeneration Biomacromolecules 2008 9 349 354 18067266 10.1021/bm7009015

[25] Stephan T Dubas PK Coating of polyelectrolyte multilayer thin films on nanofibrous scaffolds to improve cell adhesion J. Appl. Polymer Sci 2009 114 1574 1579

